# 606. Efficacy and Safety of Ceftazidime-Avibactam Comparative Dosage Regimens in Patients with Kidney Injury: A Retrospective Cohort Study

**DOI:** 10.1093/ofid/ofac492.658

**Published:** 2022-12-15

**Authors:** Walaa Adel Sait, Nader Damfu, Doaa Aljefri, Sherine Esmail, Hani Alhamdan

**Affiliations:** King Abdulaziz Medical City, Jeddah, Makkah, Saudi Arabia; King AbdulAziz medical city, Jeddah, Makkah, Saudi Arabia; King AbdulAziz medical city, Jeddah, Makkah, Saudi Arabia; King AbdulAziz medical city, Jeddah, Makkah, Saudi Arabia; King AbdulAziz medical city, Jeddah, Makkah, Saudi Arabia

## Abstract

**Background:**

Limited data are available for Ceftazidime-avibactam (CAZ AVI) dosing in patients with chronic kidney diseases. High rate of treatment failure was found among patients with chronic kidney diseases for which renally adjusted doses has been used^(1)^. In our institution, higher than labeled adjusted dosing regimens (HLAD) has been used for the treatment of severe infections. Doses were driven from ceftazidime adjusted dosing regimens in CKD patients. Therefore, we aimed to compare the efficacy and safety between labeled and HLAD regimens of CAZ-AVI in CKD patients.

**Methods:**

A retrospective cohort study conducted between June 2018 and December 2020 in a tertiary hospital in Saudi Arabia, Jeddah. Patients ≥ 14 years old who received at least 48 hours of CAZ-AVI and have chronic kidney disease or on dialysis were included. The primary outcome was to assess the clinical cure of the HLAD group compared to labeled adjusted doses (LAD) group and defined as complete or partial resolution of fever and leukocytosis at the end of CAZ-AVI course. The secondary outcome was 30-day-mortality secondary to infections and recurrent of infections. Fisher's exact, chi-square test and contingency table analysis were used as appropriate.

**Results:**

A total of 75 patients were included, 40 patients (46.7%) received HLAD. Fifty percent of the patients were on hemodialysis during CAZ-AVI course (n=38, 50%). The majority of our patients were in ICU (n=48, 64%). Clinical cure was relatively the same among the two groups with no statistical difference (57% with higher dose and 66%, 0.095 (-0.31-0.12), P=0.39). The number of 30-day mortality secondary to infection was higher (n=48, 64%) among the HLAD group compared to LAD group (74.2% and 55% respectively). Recurrence of infection were low in this study, which can be explained by the high rates of mortality, however, it was higher among patients who received LAD.

**Conclusion:**

The efficacy and safety of both regimens of CAZ-AVI dosing were comparable and was not statistically significant in renally impaired patients. Larger studies are needed, however, to answer the study question on the use of higher than labeled adjusted dosing.

**Reference:**

Shields RK, Nguyen MH, Chen L, Press EG, Kreiswirth BN, Clancy CJ. Pneumonia and renal replacement therapy are risk factors for ceftazidime-avibactam treatment failures and resistance. Antimicrob Agents Chemother. 2018;62(5)

**Disclosures:**

**All Authors**: No reported disclosures.

